# NTMT1 (METTL11A) promotes MYC-dependent proliferation and downstream HLA-A suppression in cervical cancer

**DOI:** 10.3389/fimmu.2026.1853434

**Published:** 2026-07-24

**Authors:** Jinling Zhang, Chen Chen, Huibin Song, Diying Li

**Affiliations:** 1 Department of Gynecology, Shenzhen People’s Hospital (The Second Clinical Medical College, Jinan University; The First Affiliated Hospital, Southern University of Science and Technology), Shenzhen, Guangdong, China; 2 Department of Pathology, School of Basic Medical Sciences, Southern Medical University, Guangzhou, Guangdong, China; 3 Department of Thoracic Surgery, Shenzhen People’s Hospital (The First Affiliated Hospital, Southern University of Science and Technology; The Second Clinical Medical College, Jinan University), Shenzhen, Guangdong, China

**Keywords:** cervical cancer, HLA-A, MYC signaling, NTMT1 (METTL11A), tumor immune microenvironment

## Abstract

**Background:**

Identifying upstream molecular regulators linking tumor proliferation with immune-related alterations remains an important challenge in cancer therapy. NTMT1 (METTL11A), a protein N-terminal methyltransferase, has been implicated in tumorigenesis; however, its role in coordinating tumor–immune interaction is poorly understood.

**Methods:**

We performed integrative single-cell RNA sequencing (GSE208653) and spatial transcriptomics (GSE208654) analyses to characterize NTMT1 expression and function in cervical cancer. Spatial deconvolution and microenvironmental co-localization analyses were used to define context-specific effects. Functional validation was conducted using RT–qPCR, Western blotting, multiplex immunofluorescence, and flow cytometry-based assays.

**Results:**

NTMT1 exhibited heterogeneous expression across epithelial and immune cell populations, with enrichment in squamous cell carcinoma. Spatial transcriptomics revealed that NTMT1-positive regions were associated with altered immune composition, including reduced macrophage and increased NK/T cell infiltration. In epithelial-enriched regions, NTMT1 expression correlated with activation of cell cycle pathways, including MYC, E2F1, CDK1, and CCNB1. Functional experiments demonstrated that NTMT1 promotes cell cycle progression via MYC upregulation. In epithelial–NK/T co-localization niches, NTMT1 was associated with modulation of antigen presentation pathways and suppression of HLA-A expression. Functionally, NTMT1 overexpression reduced IFN-γ production by CD8^+^ T cells under the co-culture conditions used in this study, which was partially restored by HLA-A re-expression.

**Conclusion:**

NTMT1 was identified as a candidate regulator associated with MYC activation and HLA-A suppression. Although the precise molecular mechanism remains to be elucidated, functional experiments demonstrated that NTMT1 overexpression was accompanied by increased MYC expression, reduced HLA-A expression, enhanced cell-cycle progression, and impaired CD8^+^ T-cell function. These findings identify NTMT1 as a candidate regulatory node associated with MYC activation and downstream HLA-A suppression, warranting further mechanistic and *in-vivo* investigation, highlighting NTMT1 as a promising candidate for future therapeutic investigation. Further *in vivo* studies will be required to determine its suitability as a target for combination immunotherapy.

## Introduction

Cervical cancer remains a major global health burden, over 600,000 new cases and more than 340,000 deaths annually, with a disproportionate impact in low- and middle-income countries (1, 2). Despite advances in human papillomavirus (HPV) vaccination and screening, a significant proportion of patients still present with advanced or recurrent disease requiring systemic therapy (3). Recent therapeutic developments, including immune checkpoint inhibitors, have improved outcomes in selected populations; however, overall response rates remain limited and heterogeneous (4). This limited efficacy is largely attributed to the coexistence of sustained tumor proliferation and adaptive immune evasion within the tumor microenvironment (TME), where impaired antigen presentation and T cell dysfunction enable tumor persistence (5, 6). This duality underscores an urgent need to identify molecular targets associated with both oncogenic growth and immune-related phenotypes. However, current molecular targeting strategies largely fail to address this duality in an integrated manner. Most currently investigated therapeutic targets in cervical cancer are functionally restricted to either proliferative signaling pathways or immune regulatory axes. Oncogenic drivers such as MYC, E2F, and cyclin-dependent kinases (CDKs) primarily regulate cell cycle progression and tumor expansion, whereas immune checkpoint molecules, including PD-1/PD-L1 and CTLA-4, govern T cell exhaustion and immune escape (7, 8). However, accumulating evidence suggests that tumor progression is orchestrated through dynamic and reciprocal interactions between malignant cells and immune components within the TME (9). Notably, dysregulation of antigen presentation pathways, particularly alterations in major histocompatibility complex (MHC) class-I molecules such as HLA-A, has been linked to reduced CD8^+^ T cell infiltration and poor immunotherapy response (10, 11). Despite this recognition, molecular regulators that integrate tumor-intrinsic proliferation with immune modulation remain insufficiently characterized, representing a critical gap in current therapeutic strategies. Protein methylation has emerged as a crucial post-translational modification governing cancer-associated processes, including chromatin remodeling, transcriptional regulation, and protein stability (12). N-terminal methyltransferase 1 (NTMT1), also known as METTL11A, catalyzes α-N-methylation of protein substrates, thereby modulating protein interactions and functional activity (13). Despite emerging evidence linking NTMT1 to tumorigenesis, its role as a coordinated regulator of tumor proliferation and immune evasion remains undefined (14). However, while its role in tumor proliferation is increasingly recognized, its involvement in immune regulation within the TME particularly in antigen presentation and cytotoxic T-cell function remains largely unexplored. In this study, we performed integrative single-cell and spatial transcriptomic analyses combined with experimental validation to systematically characterize the role of NTMT1 in cervical cancer progression. These findings suggest that NTMT1-mediated HLA-A downregulation reduces CD8^+^ T-cell activation under the experimental conditions used in this study, supporting a potential role for NTMT1 in immune evasion. Further studies using cervical cancer antigen-specific, HLA-matched T-cell models and direct tumor-cell cytotoxicity assays will be required to establish the effects of NTMT1 on HLA-A-dependent tumor immune recognition.

## Methods

### Ethical statement

This study was conducted in accordance with the principles outlined in the Declaration of Helsinki and approved by the Institutional Review Board (IRB) of the participating institution. This study was approved by the Ethics Committee of Shenzhen People’s Hospital (Approval No. LL-KY-2025311-01). All human tissue samples used for multiplex immunofluorescence analysis were obtained from patients undergoing surgical resection, with prior written informed consent. Peripheral blood samples used for CD8^+^ T cell isolation were collected from healthy volunteers following informed consent and in compliance with institutional ethical guidelines. Publicly available datasets (GSE208653 and GSE208654) were obtained from the Gene Expression Omnibus (GEO) database. As these datasets are fully de-identified and publicly accessible, additional ethical approval was not required for their use. All experimental procedures involving human-derived materials were performed in accordance with our institutional regulations. No animal experiments were conducted in this study. HPV genotyping results were retrieved from the corresponding clinical pathology records generated during routine diagnostic evaluation. HPV status was used to characterize the clinical specimens included in this study but was not incorporated into the statistical analyses because all available specimens were positive for high-risk HPV infection.

### Data acquisition and preprocessing

Publicly available single-cell RNA sequencing (scRNA-seq) data were obtained from the Gene Expression Omnibus (GEO) https://www.ncbi.nlm.nih.gov/geo/ under accession number GSE208653, comprising cervical tissues across four pathological states: normal cervix, high-grade squamous intraepithelial lesion (HSIL), squamous cell carcinoma (SCC), and adenocarcinoma (ADC). Corresponding spatial transcriptomics data were retrieved from GSE208654, generated using the 10x Genomics Visium platform from matched tissue sections representing the same disease spectrum. All analyses were conducted in R (v4.4.1) using standardized and reproducible workflows. Single-cell and spatial transcriptomic analyses were performed using widely validated computational frameworks, including Seurat and associated Bioconductor packages, following established best-practice guidelines for data normalization, integration, and downstream analysis (15, 16). For the tumor-versus-normal comparison, bulk RNA-seq for cervical tumors (TCGA-CESC) and normal cervix (GTEx) was obtained from the UCSC Xena TOIL pan-cancer database (https://xenabrowser.net), which uniformly reprocesses TCGA and GTEx RNA-seq was compared by two-sided Mann–Whitney test.

### Single-cell RNA sequencing analysis

Raw scRNA-seq data were processed using the Seurat package (v5.4.0) following established best-practice workflows for single-cell transcriptomic analysis (15, 16). Cells were first subjected to stringent quality control to remove low-quality cells and potential doublets based on gene count of more than 500 and less than 8000, and mitochondrial gene content of less than 25%. Data normalization, variance stabilization, and regression of confounding sources of variation was performed using the SCTtransform with default parameters Dimensionality reduction was conducted using principal component analysis (PCA) with 50 components, and the top 30 principal components were used for downstream analyses. Non-linear dimensionality reduction and visualization were performed using Uniform Manifold Approximation and Projection (UMAP) with 30 dimensions as input. Cell clustering was carried out using a shared nearest neighbor (SNN) graph-based modularity optimization algorithm implemented in Seurat at resolution of 0.1, resulting in 15 clusters which were annotated into 10 major cell types. Cell type annotation was performed based on the expression of canonical marker genes, including EPCAM (epithelial cells), MS4A1 (B cells), LYZ and SORL1 (macrophages), VWF (endothelial cells), PDGFRA (fibroblasts), CPA3 (mast cells), and JCHAIN (plasma cells), CD3D and NKG7 (NK/T cells), CSF3R (neutrophils), MYH11 (smooth muscle cells). Annotations were further validated by examining the distribution and specificity of marker gene expression across clusters to ensure consistency with established cell lineage signatures.

### Spatial transcriptomics deconvolution

To infer cell type composition within spatial transcriptomic spots, we applied SPOTlight (v1.10.0), a seeded non-negative matrix factorization (NMF)-based deconvolution method that leverages cell type–specific transcriptional signatures derived from single-cell RNA sequencing data (17). The scRNA-seq dataset was converted into a *SingleCellExperiment* object to ensure compatibility with downstream Bioconductor workflows. Prior to feature selection, ribosomal protein genes (RPL/RPS) and mitochondrial genes (MT-) were excluded to minimize technical bias. Highly variable genes (n = 3,000) were identified using the *scran* package via the *modelGeneVar* function (Lun et al., 2016). Cell type–specific marker genes were ranked using the *scoreMarkers* function, and features with mean area under the curve (AUC) > 0.8 were retained to ensure high discriminatory power. To reduce computational complexity while preserving representative transcriptional diversity, 50 cells per annotated cell type were randomly sampled to construct the reference matrix. Deconvolution was performed on SCTransform-normalized spatial transcriptomics data using default SPOTlight parameters, generating a probabilistic estimate of cell type proportions for each spatial spot. The resulting cell type proportion matrix was integrated into the Seurat spatial object metadata and used for downstream analyses, including spatial cell type mapping, co-localization analysis, and comparative assessment between NTMT1-positive and NTMT1-negative regions.

### METTL11A expression stratification and spatial co-localization analysis

Spatial spots were classified based on NTMT1 (METTL11A) expression. Spots with normalized expression > 0 were defined as NTMT1-positive, whereas those with zero expression were classified as NTMT1-negative. To investigate microenvironmental context, co-localization regions were defined within SCC samples using SPOTlight-derived cell proportions. Two biologically relevant niches were identified: (i) epithelial–macrophage (Epi ≥15%, macrophage ≥15%) and (ii) epithelial–NK/T cell (Epi ≥15%, NK/T ≥15%). These thresholds ensured biologically meaningful co-occurrence while preserving statistical power. The detection-based threshold (>0) was adopted because, in Visium spatial data, the per-spot capture depth for a low-abundance transcript such as NTMT1 is shallow and the count distribution is dominated by 0 and 1; the informative contrast is therefore “detected vs not detected”. Robustness of the key findings to this cutoff was confirmed under six stricter definitions of positivity (**Supplementary Table 1**). The 15% co-localization cut-off was justified by a spatial-independence permutation test (1,000 permutations: Epi–NK/T observed 487 vs null 607.6, z = −12.7, two-sided p ≈ 5×10^−37^), and to balance cellular co-occurrence stringency against an adequate number of spots for differential testing: lower thresholds (10%) admit more spots but weaker co-occurrence, while higher thresholds (>=20%) are too sparse for statistical power (n=98 at 20%).

### Differential expression and functional enrichment analysis

Differential expression analysis between NTMT1-positive and NTMT1-negative spots was performed using Seurat (FindMarkers, Wilcoxon rank-sum test). Genes expressed in fewer than 10% of spots were excluded (min.pct = 0.1), and a log fold-change threshold of 0.1 was applied. *P*-values were adjusted using the Benjamini–Hochberg method, with adjusted *p* < 0.05 considered significant. Genes with |log_2_FC| > 0.25 were used for downstream analyses. Functional enrichment was performed using clusterProfiler (v4.14.6). Gene Ontology (GO) and KEGG analyses were conducted with adjusted *p* < 0.05 and q < 0.2. Additional pathway enrichment was performed using EnrichR across KEGG, Reactome, WikiPathways, and GO databases. Gene set enrichment analysis (GSEA) was conducted using ranked gene lists and MSigDB gene sets via the msigdbr package. Gene identifiers were converted using org.Hs.eg.db.

### Multiplex immunofluorescence staining

Formalin-fixed, paraffin-embedded (FFPE) tissue sections (4 μm thickness) were deparaffinized in xylene and rehydrated through a graded ethanol series. Antigen retrieval was performed in Tris–EDTA buffer (pH 9.0) at 110 °C for 15 min, followed by cooling to room temperature. Endogenous peroxidase activity was quenched prior to staining. Multiplex immunofluorescence staining was performed using the PANO 6-plex IHC kit (Panovue, Beijing, China) according to the manufacturer’s instructions, employing a sequential staining and stripping protocol. Briefly, primary antibodies against METTL11A, MYC, and HLA-A were applied sequentially, each followed by horseradish peroxidase (HRP)-conjugated secondary antibodies and tyramide signal amplification (TSA)-based fluorophore deposition. After each staining cycle, antibody stripping was performed to enable subsequent marker detection without signal overlap. Nuclei were counterstained with DAPI, and stained slides were mounted using antifade medium. Whole-slide imaging was performed using an Olympus VS200 slide scanner under identical exposure and acquisition settings to ensure comparability across samples. Quantitative image analysis was conducted using inForm software (v2.4, PerkinElmer). Tissue segmentation and single-cell segmentation were performed based on DAPI nuclear staining and membrane features. Fluorescence intensity thresholds for each marker were defined using internal controls and applied consistently across all samples. Marker expression was quantified as the proportion of positive cells and mean fluorescence intensity within defined regions of interest.

### Gene expression and protein analysis

#### Cell culture and transfection

Human cervical squamous cell carcinoma cell line SiHa and Caski were purchased from the Cellcook (Guangzhou, China). The SiHa cell was cultured in MEM medium (Gibco, USA) with 10% fetal bovine serum (Gibco), 1% penicillin-streptomycin (Gibco) and 1% Nonessential amino acid (Cellcook) at 37 °C, 5% CO_2_. The Caski cell was cultured in RPMI 1640 (Gibco, USA) with 10% fetal bovine serum (Gibco) and 1% penicillin-streptomycin (Gibco) at 37 °C, 5% CO_2_. Lipofectamine3000 was used to transfect the siRNAs or plasmids into the cells according to the previous study (18).

### RT–qPCR analysis

Total RNA was extracted using TRIzol reagent (Invitrogen) according to the manufacturer’s instructions. RNA concentration and purity were assessed spectrophotometrically, and 1 μg of total RNA was reverse-transcribed into cDNA using the M-MLV Reverse Transcriptase Kit (Promega). Quantitative PCR was performed using the Bio-Rad iQ5 Real-Time PCR System with SYBR Green chemistry. Gene-specific primers for METTL11A, MYC, HLA-A, and GAPDH were designed using Primer-BLAST (NCBI). The primer sequences are as follows: METTL11A-PF: ATGACGAGCGAGGTGATA; METTL11A-PR: GTCGATGCTGGAGATGTG; MYC-PF: AGCCACAGCATACATCCT; MYC-PR: CGCACAAGAGTTCCGTAG; HLA-A-PF: CGACGGCAAGGATTACAT; HLA-A-PR: CCAGGTAGGCTCTCAACT. Relative gene expression levels were calculated using the 2^^−ΔΔCt^ method (19), with GAPDH serving as the internal control. All reactions were performed in triplicate, and no-template controls were included to exclude contamination.

### Western blot analysis

Cells were lysed in RIPA buffer supplemented with protease inhibitors, and protein concentrations were determined using a bicinchoninic acid (BCA) assay (20). Equal amounts of protein (20 μg per sample) were separated by SDS-PAGE and transferred onto polyvinylidene difluoride (PVDF) membranes. Membranes were blocked with 5% bovine serum albumin (BSA) in TBST for 1 h at room temperature and incubated overnight at 4 °C with primary antibodies against METTL11A, MYC, HLA-A, and GAPDH. After washing, membranes were incubated with HRP-conjugated secondary antibodies for 1 h at room temperature. Protein bands were visualized using enhanced chemiluminescence (ECL) and imaged using a Bio-Rad ChemiDoc system. Band intensities were quantified using ImageJ software and normalized to GAPDH.

### Cell cycle and immune functional assays

#### Cell cycle analysis

Cell cycle distribution was assessed by propidium iodide (PI) staining following synchronization using a double thymidine block (21). Briefly, cells were treated with 2 mM thymidine for 18 h, released into fresh medium for 9 h, and subjected to a second thymidine block for 16 h to achieve G1/S synchronization. After release, cells were harvested at the indicated time points, fixed in 70% ethanol overnight at 4 °C, and stained with PI (50 μg/mL) in the presence of RNase A (100 μg/mL) for 30 min at 37 °C in the dark. DNA content was analyzed using a BD FACSMelody flow cytometer, and at least 10,000 events per sample were acquired. Cell cycle distribution (G0/G1, S, and G2/M phases) was quantified using FlowJo software (v10) after exclusion of debris, doublets, and non-viable cells based on forward and side scatter properties.

### CD8^+^ T cell isolation and co-culture assay

Peripheral blood mononuclear cells (PBMCs) were isolated from healthy donors by Ficoll-Paque density gradient centrifugation. CD8^+^ T cells were purified by fluorescence-activated cell sorting (FACS) using anti-CD8A antibodies. For functional assays, CD8^+^ T cells were co-cultured with SiHa cells under indicated experimental conditions. To assess CD8^+^ T-cell functional activation, T cells were stimulated with CMV pp65 peptide (1 μg/mL) for 6 h in the presence of brefeldin A and monensin for intracellular cytokine analysis.

### Intracellular cytokine staining

Following stimulation, cells were fixed and permeabilized using a commercially available intracellular staining kit according to the manufacturer’s protocol. Cells were stained with fluorochrome-conjugated anti–IFN-γ antibodies and analyzed by flow cytometry. Data acquisition was performed on a BD FACSMelody cytometer, and analysis was conducted using FlowJo software (v10). CD8^+^ T cells were gated based on forward/side scatter and CD8 expression, and cytokine-producing cells were quantified as the percentage of IFN-γ^+^ cells within the CD8^+^ T cell population. Appropriate fluorescence minus one (FMO) controls were used to define gating thresholds. All flow cytometry experiments were performed with at least three independent biological replicates.

### Statistical analysis

All data are presented as mean ± standard deviation (SD). Statistical comparisons were performed using two-tailed Student’s t-test or one-way ANOVA, as appropriate. Multiple testing correction was performed using the Benjamini–Hochberg method. A two-sided *p* < 0.05 was considered statistically significant.

## Results

### Single-cell landscape reveals heterogeneous NTMT1 expression in cervical cancer

**Figure 1:**
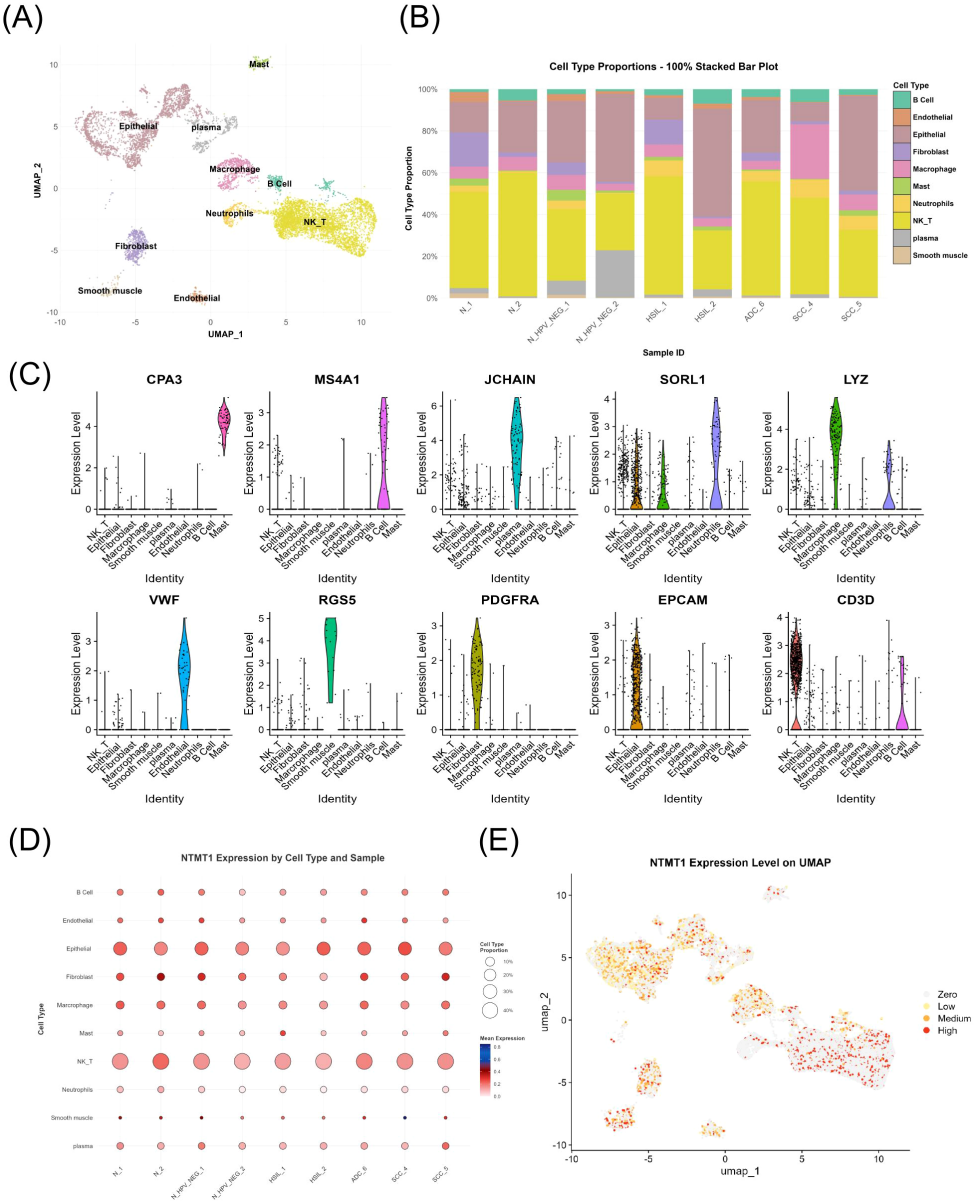
Single-cell transcriptomic profiling reveals heterogeneous NTMT1 expression across cervical cancer cell types. **(A)** UMAP visualization of scRNA-seq data from cervical tissues (GSE208653), showing distinct clustering of major cell populations, including epithelial, macrophage, NK/T, B cell, endothelial, fibroblast, mast, neutrophil, plasma, and smooth muscle cells; **(B)** Stacked bar plot illustrating the proportional distribution of cell types across different disease conditions (Normal, HSIL, ADC, and SCC), highlighting dynamic changes in epithelial, immune, and stromal compartments during disease progression; **(C)** Violin plots showing expression of canonical marker genes used for cell type annotation across identified clusters, confirming the specificity of cell type identities; **(D)** Dot plot depicting NTMT1 expression across cell types and samples. Dot size represents the proportion of NTMT1-expressing cells, and color intensity indicates average expression levels; **(E)** UMAP feature plot showing the distribution of NTMT1 expression across all cells, with expression levels categorized as zero, low, medium, and high.

To delineate the cellular landscape of cervical cancer and characterize NTMT1 expression at single-cell resolution, we analyzed scRNA-seq data from normal, HSIL, SCC, and ADC samples (GSE208653). Unsupervised clustering identified distinct cellular populations spanning epithelial, immune, and stromal compartments, which were visualized using UMAP embedding (**Figure 1A**). Major cell types included epithelial cells, macrophages, NK/T cells, B cells, endothelial cells, fibroblasts, mast cells, neutrophils, plasma cells, and smooth muscle cells, consistent with known cervical tumor microenvironment composition. Comparative analysis of cellular composition across disease states revealed dynamic remodeling of the tumor microenvironment during disease progression (**Figure 1B**). Epithelial cells constituted a dominant population across all samples, while immune cell subsets including NK/T cells and macrophages showed variable enrichment across pathological conditions, suggesting stage-specific immune modulation. Cell type annotation was validated using canonical marker genes. Distinct and cell type–specific expression patterns were observed, including EPCAM in epithelial cells, MS4A1 in B cells, JCHAIN in plasma cells, LYZ and SORL1 in macrophages, VWF and RGS5 in endothelial cells, and PDGFRA in fibroblasts, confirming robust cluster identity and annotation accuracy (**Figure 1C**). We next examined NTMT1 expression across cell types and disease conditions. Dot plot analysis revealed that NTMT1 is broadly expressed across multiple cell populations but exhibits cell type–specific enrichment patterns (**Figure 1D****).** Notably, higher proportions of NTMT1-expressing cells were observed in epithelial and NK/T cell populations, suggesting potential roles in both tumor-intrinsic and immune-associated processes. UMAP feature visualization further demonstrated that NTMT1 expression is heterogeneously distributed across cellular clusters, with regions of high expression localized predominantly within epithelial and immune cell compartments (**Figure 1E****).** This spatially dispersed yet cell type–enriched expression pattern indicates that NTMT1 may function at the interface of tumor proliferation and immune regulation. Present findings establish a comprehensive single-cell atlas of cervical cancer and identify NTMT1 as a widely expressed but heterogeneously regulated molecule, with preferential enrichment in epithelial and immune compartments, supporting its potential role as a mediator of tumor–immune interactions.

### Spatial transcriptomics reveals NTMT1-associated remodeling of the tumor immune microenvironment

**Figure 2:**
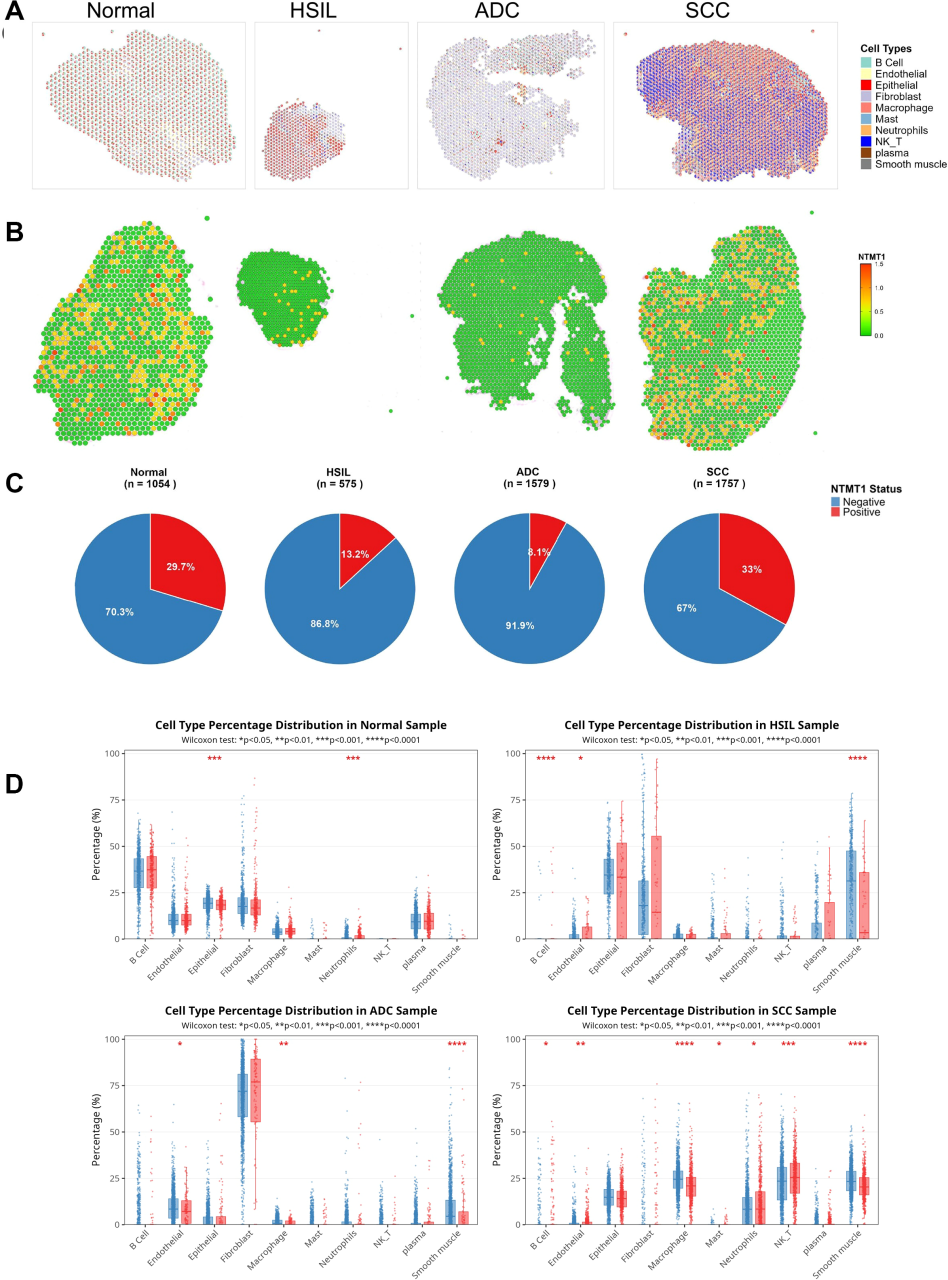
Spatial transcriptomics reveals NTMT1-associated remodeling of the tumor immune microenvironment. **(A)** Spatial maps of predicted cell type composition across Normal, HSIL, ADC, and SCC tissue sections based on SPOTlight deconvolution. Each spot is colored according to its dominant cell type; **(B)** Spatial distribution of NTMT1 expression across tissue sections. Color intensity indicates normalized expression levels, highlighting heterogeneous spatial patterns; **(C)** Pie charts showing the proportion of NTMT1-positive and NTMT1-negative spots across disease conditions. SCC exhibits the highest proportion of NTMT1-positive spots; **(D)** Comparison of cell type proportions between NTMT1-positive and NTMT1-negative regions across tissue types. Statistical significance was assessed using the Wilcoxon rank-sum test with multiple testing correction. *p<0.05, ** p<0.001 and *** p<0.0001.

To investigate the spatial organization of NTMT1 expression and its association with the tumor microenvironment, we analyzed spatial transcriptomics data across Normal, HSIL, ADC, and SCC cervical tissues (GSE208654). Cell type deconvolution using SPOTlight revealed well-defined spatial architecture, with distinct epithelial, immune, and stromal compartments across all tissue types (**Figure 2A**). Notably, SCC samples exhibited a more complex and heterogeneous cellular composition compared to precursor lesions, consistent with progressive microenvironmental remodeling during tumor development. Spatial mapping of NTMT1 expression demonstrated marked heterogeneity across tissue sections, with variable expression intensity and distribution patterns across disease states (**Figure 2B**). While NTMT1 expression was broadly detectable, regions of elevated expression were more prominent in SCC compared to HSIL and ADC, suggesting stage-dependent regulation. To quantitatively assess NTMT1 distribution, spatial spots were classified into NTMT1-positive and NTMT1-negative groups. The proportion of NTMT1-positive spots varied substantially across disease conditions (**Figure 2C**). SCC exhibited the highest proportion of NTMT1-positive spots (33%), followed by normal tissue (29.7%), whereas HSIL (13.2%) and ADC (8.1%) showed markedly lower proportions. These findings indicate that NTMT1 expression is dynamically regulated during cervical cancer progression, with enrichment in invasive disease. We next examined whether NTMT1 expression was associated with alterations in local cellular composition. Comparative analysis of cell type proportions between NTMT1-positive and NTMT1-negative regions revealed significant microenvironmental differences across all tissue types (**Figure 2D**). In SCC, NTMT1-positive regions were characterized by a reduction in macrophage proportions and a relative increase in NK/T cell infiltration, suggesting a shift in immune landscape. Similar trends, although less pronounced, were observed in other disease states. This shift toward NK/T cell enrichment alongside reduced macrophage presence suggests a reprogrammed immune microenvironment potentially linked to altered immune surveillance dynamics. Notably, the NTMT1-positive proportion in normal tissue (29.7%) was comparable to that in SCC (33%); NTMT1 is therefore best regarded not as a tumor-specific marker but as a context-dependent functional regulator. At the bulk population level (TCGA + GTEx; 304 tumors vs 13 normal cervix), NTMT1 was only modestly higher in tumor (~1.45-fold, Mann–Whitney p = 4.2×10^−4^), consistent with broad expression and modest average elevation rather than tumor-restriction (**Supplementary Figure 1**). With respect to the NTMT1-positive immune shift, the macrophage depletion was robust across all positivity thresholds (**Supplementary Table 1**), whereas the NK/T increase was threshold-sensitive and is interpreted more cautiously. The principal findings therefore remained robust to alternative NTMT1 positivity thresholds, although the magnitude of NK/T-cell enrichment and MYC-associated changes decreased under increasingly stringent cutoffs because of the reduced number of NTMT1-positive spots (**Supplementary Table 1**). This increased NK/T infiltration is checkpoint-restrained rather than simply enhanced immunity. To reconcile the increased NK/T infiltration with the immune-evasion model, we examined T-cell functional states within the epithelial–NK/T region. NTMT1-positive spots showed coordinated up-regulation of both effector markers (PRF1 log_2_FC 0.12, p=0.019; IFNG p=0.0088; CD8A p=0.043) and inhibitory checkpoints (PD-1/PDCD1 p=3.9×10^−4^, padj=0.006; LAG3 p=0.020), the signature of recruited but checkpoint-restrained lymphocytes (**Supplementary Table 2**). Thus, the increased NK/T-cell infiltration may indicate checkpoint-restrained immune infiltration rather than a straightforward enhancement of immunity. To further characterize the functional state of infiltrating NK/T cells, we analyzed the epithelial–NK/T co-localization region. NTMT1-positive regions showed increased expression of cytotoxic and effector genes (PRF1, IFNG, and CD8A) together with inhibitory checkpoint molecules (PDCD1 and LAG3), consistent with a checkpoint-restrained immune phenotype rather than fully effective anti-tumor immunity (**Supplementary Table 2**). Although aggregate cytotoxicity and exhaustion module scores did not differ significantly between groups, the differential expression of individual checkpoint and effector genes supported this interpretation.

### NTMT1 promotes MYC-driven cell cycle activation in epithelial tumor regions

**Figure 3:**
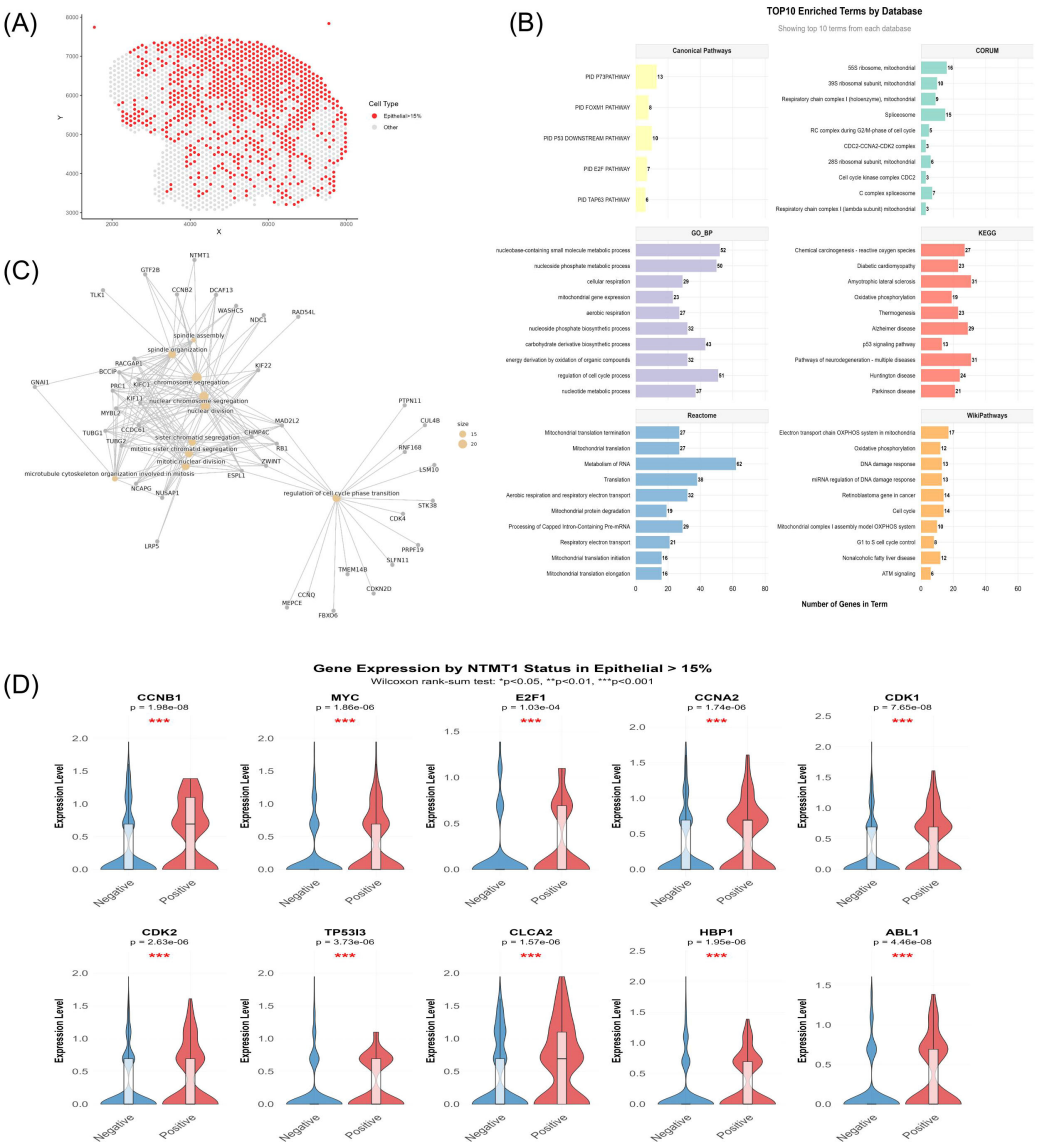
NTMT1 expression is associated with cell cycle activation in epithelial-enriched tumor regions. **(A)** Spatial identification of epithelial-enriched regions (epithelial proportion ≥15%) within SCC tissue sections. Spots are classified based on epithelial content for downstream analysis; **(B)** Top enriched pathways identified from differentially expressed genes between NTMT1-positive and NTMT1-negative epithelial regions across multiple databases (Canonical Pathways, GO, KEGG, Reactome, WikiPathways, CORUM), highlighting enrichment of cell cycle and metabolic processes; **(C)** Pathway–gene association network showing the relationships between enriched biological processes and representative differentially expressed genes in NTMT1-positive epithelial regions. The network was generated using the clusterProfiler package; **(D)** Violin plots comparing expression levels of key cell cycle genes between NTMT1-positive and NTMT1-negative epithelial regions. Statistical significance was assessed using the Wilcoxon rank-sum test. ***p<0.0001.

To investigate the functional consequences of NTMT1 expression within specific microenvironmental contexts, we focused on epithelial-enriched regions in SCC tissue sections. Spatial mapping identified regions with high epithelial cell proportions (≥15%), within which NTMT1-positive and NTMT1-negative spots were stratified for downstream analysis (**Figure 3A**). Differential expression and multi-database functional enrichment analyses revealed that NTMT1-positive regions were strongly associated with cell cycle and proliferative signaling pathways (**Figure 3B**). Canonical pathway analysis identified enrichment of key regulators, including FOXM1, E2F, PLK1, and Aurora B signaling pathways, all of which are central to cell cycle progression. Gene Ontology and Reactome analyses further highlighted enrichment of processes related to mitotic division, chromosome segregation, and mitochondrial metabolic activity, indicating coordinated activation of proliferative and bioenergetic programs. The gene-concept network analysis of differentially expressed genes demonstrated a highly interconnected module of cell cycle regulators, including CCNB1, CDK1, E2F1, and MYC, suggesting that NTMT1-associated transcriptional changes converge on a central proliferative network (**Figure 3C**). The tight connectivity of these genes supports a coordinated regulatory mechanism driving cell cycle progression. To validate these findings at the gene level, we examined the expression of key cell cycle regulators in NTMT1-positive versus NTMT1-negative epithelial regions. Multiple core regulators of cell cycle progression were significantly upregulated in NTMT1-positive spots, including CCNB1, MYC, E2F1, CCNA2, CDK1, CDK2, and ABL1 (**Figure 3D**). The consistent upregulation of these genes across independent epithelial regions indicates that NTMT1 expression is robustly associated with enhanced proliferative activity. Notably, MYC, a master regulator of cell growth and metabolism—emerged as a central node linking NTMT1 expression to downstream cell cycle activation. These findings suggest that NTMT1 may promote tumor progression through MYC-driven transcriptional programs, thereby enhancing cell cycle progression within epithelial tumor compartments. We found that NTMT1 expression is functionally associated with activation of cell cycle and proliferative signaling pathways, particularly within epithelial-enriched tumor regions, supporting its role as a driver of tumor growth.

### NTMT1 modulates HLA-dependent antigen presentation and immune signaling

**Figure 4:**
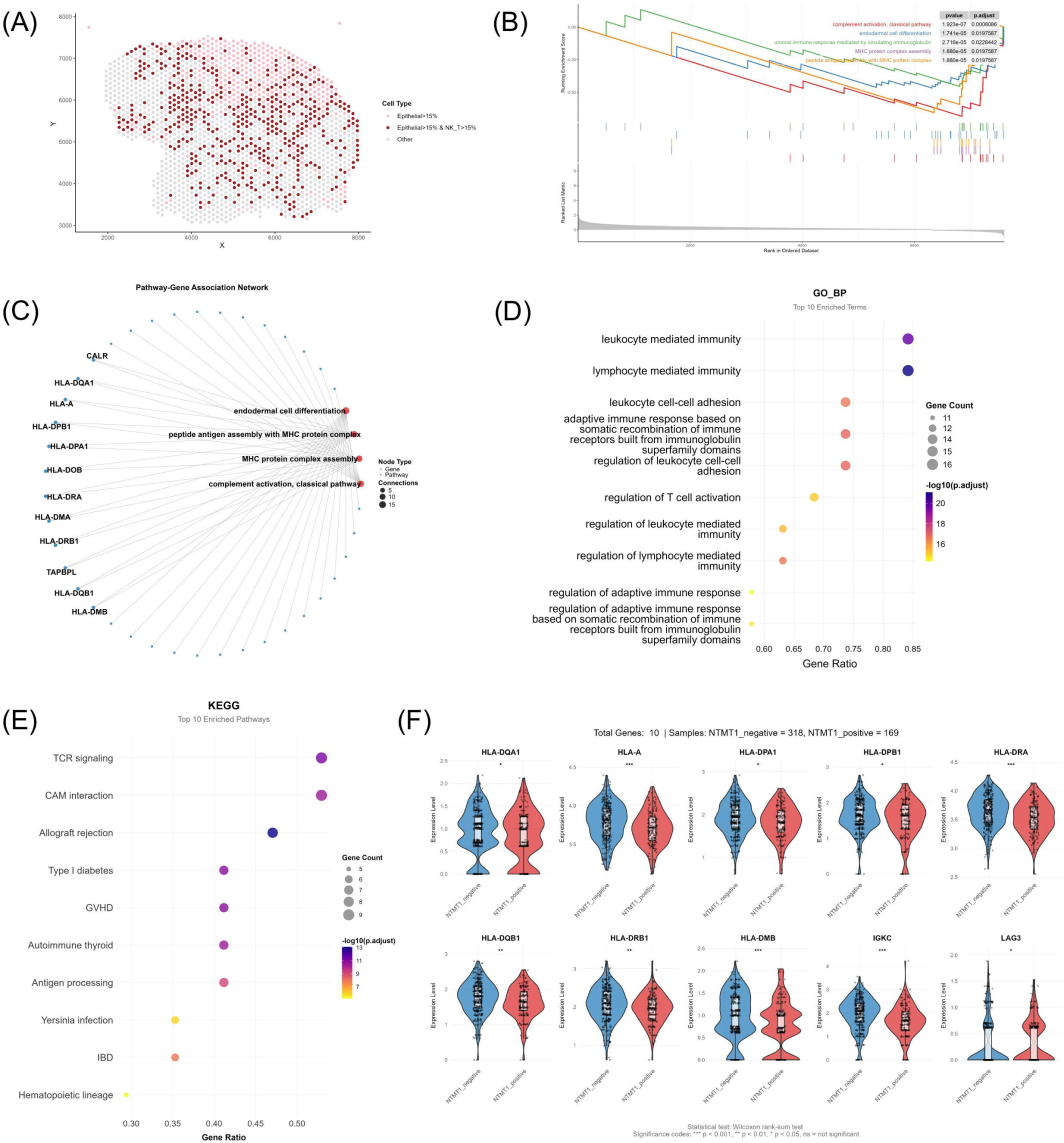
NTMT1 expression is associated with altered immune signaling in epithelial NK/T-cell co-localization regions. **(A)** Spatial identification of epithelial–NK/T cell co-localization regions (Epi ≥15% and NK/T ≥15%) within SCC tissue sections; **(B)** Gene Set Enrichment Analysis (GSEA) showing enrichment of immune-related pathways in NTMT1-positive regions, including complement activation and MHC-related processes; **(C)** Pathway–gene association network highlighting HLA family genes and their connections to antigen presentation and immune pathways; **(D)** GO Biological Process enrichment analysis of differentially expressed genes, showing enrichment in immune-related functions such as leukocyte-mediated immunity and T cell activation; **(E)** KEGG pathway enrichment analysis highlighting immune-related pathways, including T cell receptor signaling and antigen processing and presentation; **(F)** Violin plots comparing expression levels of HLA genes and immune-related markers between NTMT1-positive and NTMT1-negative regions. Statistical significance was assessed using the Wilcoxon rank-sum test. *p<0.05, ** p<0.001 and *** p<0.0001.

To investigate the immune regulatory role of NTMT1 within spatially defined microenvironments, we focused on epithelial–NK/T cell co-localization regions (Epi ≥15% and NK/T ≥15%) in SCC tissue sections (**Figure 4A**). These regions represent active tumor–immune interaction niches and provide a relevant context for assessing NTMT1-associated immune modulation. Gene set enrichment analysis (GSEA) of differentially expressed genes revealed significant enrichment of immune-related pathways in NTMT1-positive regions (**Figure 4B**). Notably, pathways associated with complement activation, MHC protein complex assembly, and peptide antigen presentation were among the top enriched processes, indicating a strong association between NTMT1 expression and antigen processing machinery. To further delineate the molecular network underlying these observations, pathway–gene association analysis identified a tightly connected module centered on HLA family genes, including HLA-A, HLA-DQA1, HLA-DPA1, HLA-DPB1, HLA-DRA, HLA-DRB1, HLA-DMB, and HLA-DQB1 (**Figure 4C**). These genes were linked to key immune processes such as MHC complex assembly, antigen presentation, and complement activation, suggesting coordinated regulation of adaptive immune pathways. Consistent with these findings, Gene Ontology analysis demonstrated significant enrichment of biological processes related to leukocyte-mediated immunity, lymphocyte activation, and regulation of T cell responses (**Figure 4D**). KEGG pathway analysis further highlighted enrichment in T cell receptor signaling, antigen processing and presentation, and allograft rejection pathways (**Figure 4E**), reinforcing the involvement of NTMT1 in shaping immune signaling networks. At the gene level, differential expression analysis revealed significant alterations in multiple HLA genes and immune regulators between NTMT1-positive and NTMT1-negative regions (**Figure 4F**). Notably, expression of key antigen presentation molecules, including HLA-A and HLA class II genes, was significantly modulated, alongside immune-related genes such as IGKC and the immune checkpoint molecule LAG3. These results demonstrate that NTMT1 expression is strongly associated with reprogramming of antigen presentation pathways and immune signaling networks within epithelial–NK/T cell niches. These findings suggest that NTMT1 may influence tumor–immune interactions by modulating HLA-dependent antigen presentation and downstream T cell responses.

### NTMT1 correlates positively with MYC and inversely with HLA-A in cervical cancer

**Figure 5:**
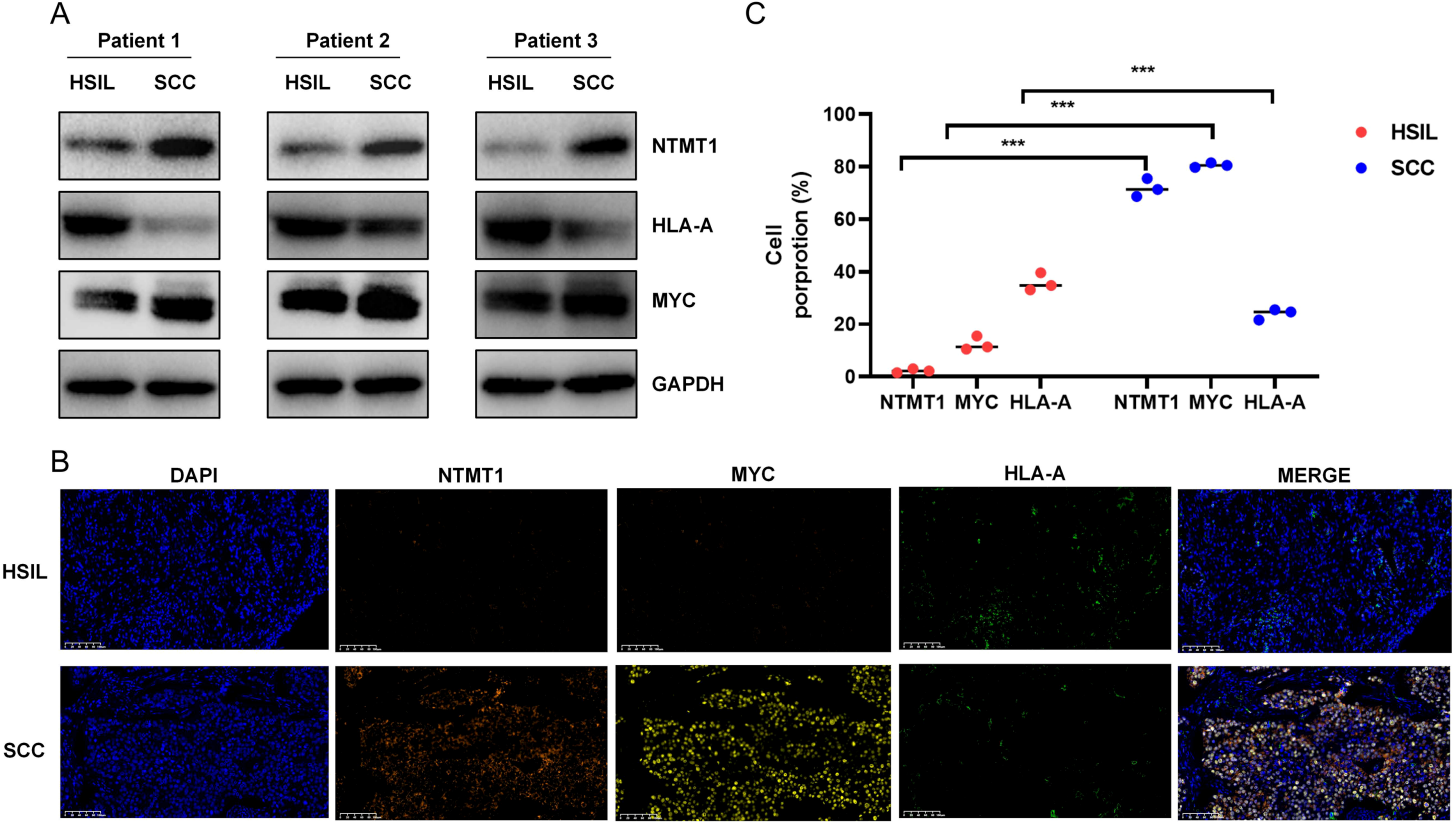
NTMT1 expression correlates with MYC upregulation and HLA-A downregulation in cervical cancer tissues. **(A)** Western blot analysis of METTL11A, MYC, and HLA-A protein expression in HSIL and SCC tissues, with GAPDH as a loading control; **(B)** Multiplex immunofluorescence staining of METTL11A, MYC, and HLA-A in HSIL and SCC tissue sections. Representative images show increased METTL11A and MYC expression and decreased HLA-A expression in SCC. Nuclei are stained with DAPI. Scale bar, 100 μm; **(C)** Quantitative analysis of METTL11A-, MYC-, and HLA-A-positive cell proportions in HSIL and SCC tissues. Data are presented as mean ± SD. Statistical significance was assessed using a two-tailed Student’s t-test (***p < 0.0001).

To validate the bioinformatics findings at the protein level, we assessed the expression of NTMT1 (METTL11A), MYC, and HLA-A in cervical precancerous lesions (HSIL) and squamous cell carcinoma (SCC) tissues. Western blot analysis demonstrated that METTL11A and MYC protein levels were markedly increased in SCC compared to HSIL, whereas HLA-A expression was significantly reduced in SCC tissues (**Figure 5A**). These findings indicate a coordinated expression pattern in which NTMT1 is positively associated with MYC activation and inversely associated with antigen presentation machinery. To further validate these observations *in situ*, multiplex immunofluorescence staining was performed on tissue sections. Consistent with the Western blot results, SCC samples exhibited a higher proportion of METTL11A- and MYC-positive cells, while HLA-A expression was markedly decreased compared to HSIL tissues (**Figure 5B**). Spatially, METTL11A and MYC signals were predominantly localized within tumor epithelial regions, whereas HLA-A expression was comparatively diminished. Quantitative analysis confirmed these trends, demonstrating a significant increase in METTL11A and MYC-positive cell proportions in SCC, accompanied by a significant reduction in HLA-A-positive cells (**Figure 5C**). The experimental validation provided that, NTMT1 expression is positively correlated with MYC activation and negatively correlated with HLA-A expression in cervical cancer, supporting a hierarchical NTMT1–MYC–HLA-A regulatory relationship.

### NTMT1 drives cell cycle progression via MYC-dependent signaling

**Figure 6:**
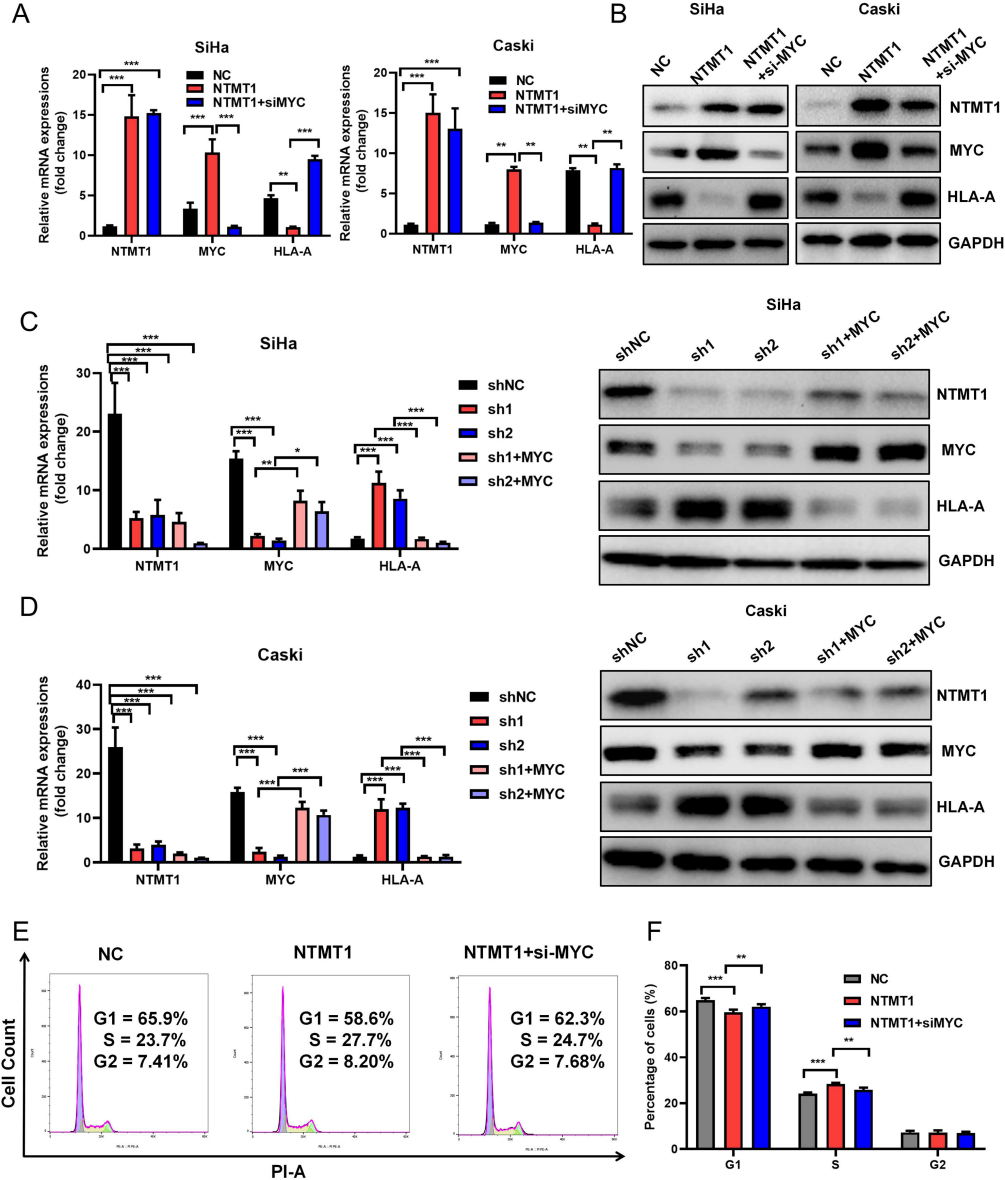
NTMT1 promotes cell cycle progression through MYC-dependent mechanisms. **(A)** RT–qPCR analysis showing METTL11A and MYC mRNA expression levels in control (NC), METTL11A-overexpressing, and METTL11A + si-MYC cells; **(B)** Western blot analysis of METTL11A and MYC protein expression under the indicated conditions, with GAPDH as a loading control; **(C)** Flow cytometry histograms showing cell cycle distribution (G1, S, and G2 phases) following METTL11A overexpression and MYC knockdown; **(D)** Quantitative analysis of cell cycle phase distribution, demonstrating increased S phase and reduced G1 phase in METTL11A-overexpressing cells, which is partially reversed by MYC silencing. *p<0.05, ** p<0.001 and *** p<0.0001.

To validate the role of NTMT1 in cervical cancer, we performed functional assays in two independent HPV-positive cervical cancer cell lines (SiHa and CaSki) following NTMT1 (METTL11A) overexpression with or without MYC silencing. RT–qPCR analysis demonstrated that NTMT1 overexpression significantly increased NTMT1 mRNA expression and was accompanied by a marked increase in MYC mRNA levels in both cell lines (**Figure 6A**). Importantly, MYC silencing in NTMT1-overexpressing cells markedly reduced MYC expression without affecting NTMT1 expression, supporting the conclusion that MYC functions downstream of NTMT1. Consistent with the transcriptional findings, Western blot analysis showed that NTMT1 overexpression was associated with increased MYC protein levels in both SiHa and CaSki cells, whereas MYC knockdown effectively reversed this increase (**Figure 6B**). We next examined the functional consequences of the NTMT1–MYC regulatory axis on cell-cycle progression. Flow cytometry analysis revealed that METTL11A overexpression resulted in a significant decrease in G1 phase cells and a corresponding increase in S phase cells, indicating enhanced cell cycle progression (**Figures 6C, D**). Notably, MYC silencing in METTL11A-overexpressing cells partially reversed this phenotype, restoring G1 phase accumulation and reducing S phase entry. Quantitative analysis confirmed that the METTL11A-induced shift toward S phase was significantly attenuated upon MYC knockdown, demonstrating that MYC is required for NTMT1-mediated cell cycle progression (**Figure 6D**). These findings indicate that NTMT1 overexpression is associated with a modest but reproducible enhancement of MYC-dependent cell-cycle progression. Although the magnitude of the cell-cycle shift was moderate, the results were consistent with the transcriptomic analyses and molecular changes observed in MYC expression, supporting a role for NTMT1 in regulating proliferative signaling.

### NTMT1 impairs CD8^+^ T-cell function through MYC-dependent HLA-A suppression

**Figure 7:**
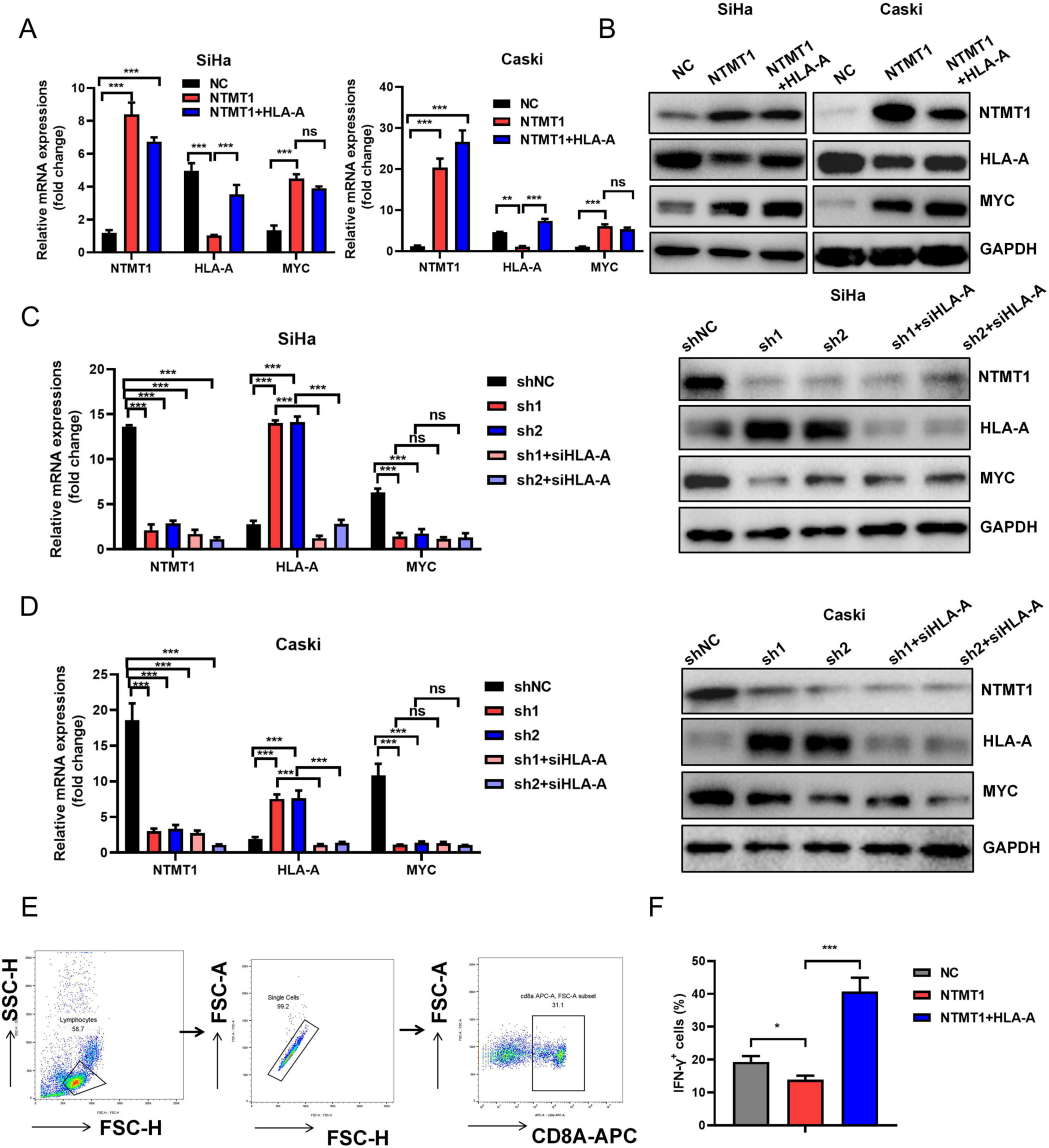
NTMT1 suppresses CD8^+^ T cell cytotoxicity through HLA-A downregulation. **(A)** RT–qPCR analysis showing HLA-A and METTL11A mRNA expression in control (NC), METTL11A-overexpressing, and METTL11A + HLA-A cells; **(B)** Western blot analysis of METTL11A and HLA-A protein expression under the indicated conditions, with GAPDH as a loading control; **(C)** Flow cytometry gating strategy for isolation of CD8^+^ T cells from peripheral blood mononuclear cells; **(D)** Schematic representation of the co-culture system used to assess CD8^+^ T cell cytotoxic function; **(E)** Quantification of IFN-γ–producing CD8^+^ T cells under different conditions. **(F)** METTL11A overexpression reduces CD8^+^ T cell function, which is restored by HLA-A re-expression. Statistical significance was assessed using Student’s t-test (*p < 0.05, ***p < 0.001).

To investigate whether NTMT1 influences tumor immune interactions through the MYC–HLA-A regulatory axis, we examined the effects of NTMT1 overexpression on HLA-A expression in two independent cervical cancer cell lines (SiHa and CaSki). RT–qPCR analysis demonstrated that NTMT1 overexpression was associated with a significant reduction in HLA-A mRNA levels, whereas enforced HLA-A expression restored HLA-A expression in NTMT1-overexpressing cells (**Figure 7A**). Importantly, HLA-A overexpression did not alter NTMT1 expression or MYC expression in either cell line, supporting the conclusion that HLA-A functions downstream of the NTMT1–MYC regulatory axis. Consistent with the transcriptional findings, Western blot analysis demonstrated that NTMT1 overexpression reduced HLA-A protein expression in both SiHa and CaSki cells, whereas HLA-A re-expression restored HLA-A protein levels without affecting MYC expression (**Figure 7B**). Together with the MYC knockdown experiments presented in **Figure 6**, these findings support a hierarchical regulatory model in which NTMT1 acts upstream of MYC, with HLA-A suppression occurring as a downstream consequence of MYC activation rather than through an independent regulatory pathway. To determine the functional consequences of HLA-A downregulation, CD8^+^ T cells were isolated from peripheral blood mononuclear cells and validated by flow cytometry (**Figure 7E**). A co-culture assay was then performed using SiHa cells to evaluate CD8^+^ T-cell functional activation. Functional analysis revealed that NTMT1 overexpression significantly reduced the proportion of IFN-γ-producing CD8^+^ T cells, indicating reduced IFN-γ production (**Figure 7F**). Importantly, restoration of HLA-A expression significantly rescued CD8^+^ T-cell function, as evidenced by increased IFN-γ production. These findings indicate that NTMT1 overexpression alters CD8^+^ T-cell functional activation under the assay conditions used in this study by promoting MYC-dependent suppression of HLA-A, thereby reducing CD8^+^ T-cell functional activation under these assay conditions. These functional observations are consistent with the transcriptomic analyses identifying altered antigen-presentation pathways in NTMT1-positive tumor regions. HPV genotyping was performed using routine clinical diagnostic assays in the Department of Pathology, Shenzhen People’s Hospital. High-risk HPV genotypes were determined according to the manufacturer’s protocol. The HPV genotyping results of all specimens included in this study are summarized in **Supplementary Table 3**.

## Discussion

This study identifies NTMT1 as a previously unrecognized candidate upstream regulator associated with MYC-dependent proliferation and downstream HLA-A suppression. By combining single-cell and spatial transcriptomics with functional validation, we demonstrate that NTMT1 coordinates MYC-driven cell cycle activation and HLA-A–mediated suppression of anti-tumor immunity, thereby addressing a critical gap in current cancer therapeutics, where most targets selectively modulate either oncogenic growth or immune escape, but rarely both. Recent advances in cancer biology emphasize that effective therapeutic targets must operate at the interface of tumor-intrinsic and microenvironmental processes (22). While canonical oncogenic drivers such as MYC, E2F, and CDK networks are well-established regulators of proliferation, and immune checkpoints such as PD-1/PD-L1 govern immune evasion (23, 24), few molecular nodes integrate these two axes simultaneously. Our findings suggest NTMT1 may represent one such upstream node, thereby aligning with emerging efforts to identify multifunctional therapeutic targets capable of reshaping both tumor growth and immune responses. A key observation from our study is the spatially heterogeneous and compartment-specific expression of NTMT1, with enrichment in epithelial tumor cells and immune-interacting niches. This finding is consistent with recent spatial transcriptomics studies demonstrating that tumor progression is governed by cellular ecosystems rather than isolated pathways (25, 26). Unlike traditional bulk analyses, our data reveal that NTMT1 is preferentially active within epithelial–immune interaction zones, particularly regions enriched with NK/T cells, suggesting a context-dependent role in modulating tumor–immune crosstalk. This spatial specificity strengthens the biological relevance of NTMT1 as a context-dependent functional regulator within tumor–immune interaction niches. Rather than acting as a tumor-specific marker, NTMT1 appears to influence biological processes associated with MYC-dependent proliferative signaling and HLA-A-associated immune modulation within the cervical cancer microenvironment.

Mechanistically, our data establish a robust link between NTMT1 and MYC-dependent proliferative signaling. Although NTMT1-positive regions exhibited increased NK/T-cell infiltration, this finding should not be interpreted as evidence of enhanced anti-tumor immunity. Instead, additional analyses demonstrated coordinated upregulation of immune checkpoint molecules (PDCD1 and LAG3) together with cytotoxic effector genes (PRF1, IFNG, and CD8A), consistent with a state of checkpoint-restrained immune activation. These findings suggest that NTMT1-positive tumors are characterized by increased immune-cell infiltration accompanied by functional immune restraint, providing a plausible explanation for the coexistence of immune infiltration and immune evasion within the cervical cancer microenvironment. MYC is widely recognized as a central oncogenic driver that regulates cell cycle progression, ribosomal biogenesis, and metabolic reprogramming (27, 28). However, direct targeting of MYC remains challenging due to its “undruggable” structure. In this context, identifying upstream regulators of MYC has become a major focus in cancer therapy development. Our findings demonstrate that NTMT1 upregulates MYC at both transcriptional and protein levels, and that MYC silencing abrogates NTMT1-induced cell cycle progression, thereby establishing a functional dependency of NTMT1 on MYC signaling. This suggests that NTMT1 may act as an epigenetic facilitator of MYC activation, offering an alternative therapeutic entry point for targeting MYC-driven cancers. Although the observed changes in cell-cycle distribution were modest in magnitude, they were reproducible and accompanied by consistent alterations in MYC expression and cell-cycle-associated transcriptional programs identified by both transcriptomic and molecular analyses. Therefore, we interpret the flow cytometry findings as supporting a role for NTMT1 in MYC-dependent proliferative signaling rather than as evidence that NTMT1 is a dominant regulator of cell-cycle progression. As NTMT1 is an α-N-terminal protein methyltransferase rather than a DNA-binding transcription factor, our findings should be interpreted as demonstrating a functional association between NTMT1 expression and altered MYC and HLA-A expression rather than direct transcriptional regulation. Although NTMT1 overexpression consistently increased MYC expression and decreased HLA-A expression in our experimental models, the molecular intermediates linking its enzymatic activity to these transcriptional changes remain undefined. Therefore, our data support a functional NTMT1–MYC–HLA-A axis but do not establish direct biochemical regulation. Future studies employing catalytic-inactive NTMT1 mutants, quantitative N-terminal methyl-proteomics, substrate identification, and rescue experiments will be required to identify the methylated substrates and signaling intermediates responsible for these downstream effects (29). This hypothesis is consistent with emerging evidence that epigenetic modifiers can indirectly control oncogenic transcriptional programs, thereby expanding the repertoire of druggable targets upstream of MYC.

Beyond proliferation, our study reveals a potential role of NTMT1 in modulating antigen-presentation-associated immune responses, which significantly enhances its therapeutic relevance. Loss or downregulation of HLA class-I molecules, particularly HLA-A, is a well-established mechanism of tumor immune evasion that impairs CD8^+^ T cell recognition (30, 31). Our data demonstrate that NTMT1 suppresses HLA-A expression at both transcriptional and protein levels, associated with reduced IFN-γ production by CD8^+^ T cells in the co-culture assay. Importantly, restoration of HLA-A expression rescues T cell function, establishing a causal relationship between NTMT1 activity and immune suppression. These findings are particularly significant in the context of immunotherapy. Although immune checkpoint inhibitors have improved outcomes in multiple cancers, their efficacy in cervical cancer remains variable (10). One key determinant of response is the integrity of antigen presentation machinery. Tumors with defective HLA expression often exhibit resistance to checkpoint blockade due to impaired T cell recognition. Our data suggest that NTMT1-mediated HLA-A suppression may contribute to this resistance phenotype, and that targeting NTMT1 could restore antigen presentation and enhance immunotherapy efficacy. This positions NTMT1 as a potential sensitizer for immune checkpoint therapies, aligning with the goals of precision oncology.

Importantly, the simultaneous regulation of MYC and HLA-A by NTMT1 reflects a broader oncogenic strategy in which tumors coordinate proliferation with immune evasion. Previous studies have shown that MYC activation can suppress immune responses through multiple mechanisms, including regulation of PD-L1 and modulation of tumor metabolism (32, 33). Our findings extend this paradigm by identifying NTMT1 as an upstream regulator that links these processes, suggesting that NTMT1 may orchestrate a coordinated oncogenic program integrating growth and immune escape. From a therapeutic standpoint, NTMT1 represents a compelling candidate for next-generation molecular targeting strategies. Unlike conventional single-axis targets, NTMT1 inhibition could simultaneously impair tumor proliferation and enhance immune surveillance. This dual functionality is particularly relevant for combination therapies, where NTMT1 inhibitors could be used alongside immune checkpoint blockade or targeted therapies to achieve synergistic effects. Moreover, given its enzymatic activity, NTMT1 may be more amenable to pharmacological inhibition compared to transcription factors such as MYC. All clinical specimens analyzed in the present study were positive for high-risk HPV infection, with HPV16, HPV18, and HPV52 representing the predominant genotypes (**Supplementary Table 3**). Therefore, the molecular and functional observations reported here were obtained within a clinically relevant HPV-positive disease background. However, because all available specimens were HPV-positive, the present study was not designed to determine whether NTMT1 functions independently of HPV oncogenes or cooperatively with HPV-mediated immune evasion. Future studies incorporating HPV-negative cervical cancer models together with quantitative analyses of HPV viral load and E6/E7 expression will be required to clarify the mechanistic relationship between NTMT1 and HPV-driven cervical carcinogenesis.

While this study provides multi-layered evidence supporting NTMT1 as a dual regulator of tumor proliferation and immune evasion, several important avenues remain for further investigation. Although our findings establish a functional NTMT1–MYC–HLA-A axis, the precise molecular substrates and epigenetic mechanisms underlying NTMT1 activity require deeper characterization. Given its role in α-N-terminal methylation, future studies integrating proteomic and chromatin-based approaches will be essential to identify direct targets and define how NTMT1 regulates MYC-driven transcriptional programs and antigen presentation pathways. In addition, while *in vitro* assays enabled controlled mechanistic insights, validation in *in vivo* systems, including orthotopic and humanized models, will be critical to fully capture the complexity of tumor–immune interactions and therapeutic responses. Furthermore, prospective clinical studies are needed to determine whether NTMT1 expression correlates with patient outcomes, immunotherapy responsiveness, or resistance, thereby establishing its potential as both a therapeutic target and predictive biomarker. Importantly, these considerations represent opportunities rather than limitations, as the enzymatic nature of NTMT1 and its dual role in coordinating proliferation and immune evasion provide a strong rationale for the development of targeted inhibitors and combinatorial strategies, particularly in the context of immunotherapy. In addition, the immune functional assays performed in this study should be interpreted within the context of their experimental design. Although IFN-γ production provided evidence that NTMT1-mediated HLA-A downregulation influences CD8^+^ T-cell activation, the assay did not directly evaluate tumor-cell cytotoxicity or cervical cancer antigen-specific immune recognition. Furthermore, HLA compatibility between donor-derived CD8^+^ T cells and tumor cells was not assessed. Future studies incorporating HLA-matched donor–tumor pairs, cervical cancer antigen-specific T-cell models, and direct cytotoxicity assays will be important to define the role of NTMT1 in antigen presentation and anti-tumor immune surveillance.

## Conclusion

This study identifies NTMT1 as a candidate upstream regulatory node associated with MYC-dependent proliferation and downstream HLA-A suppression in cervical cancer. Through integrative multi-omics analyses and functional validation, we show that NTMT1 overexpression is associated with increased MYC expression, enhanced cell-cycle progression, and reduced HLA-A expression, supporting a hierarchical NTMT1–MYC–HLA-A regulatory axis linked to proliferative signaling and immune-related phenotypes. While our findings support a functional association between NTMT1 and these biological processes, they do not establish the direct molecular mechanisms by which NTMT1 enzymatic activity regulates MYC or HLA-A expression. Therefore, NTMT1 should be regarded as a candidate regulatory node whose downstream effects warrant further mechanistic investigation. Notably, the enzymatic nature of NTMT1 makes it a potential candidate for future pharmacological investigation; however, its therapeutic relevance remains to be validated in appropriate preclinical models. Future studies identifying the direct methylated substrates and signaling intermediates of NTMT1 will be essential to define its mechanism of action and evaluate its translational potential. Collectively, this work expands the repertoire of candidate molecular regulators in cervical cancer and provides a foundation for future mechanistic and translational studies of NTMT1-directed therapeutic strategies.

## Data Availability

The original contributions presented in the study are included in the article/**Supplementary Material**. Further inquiries can be directed to the corresponding author.

## Supplementary material

Supplementary Table 1 Robustness of SCC findings across NTMT1 positivity thresholds.

Supplementary Table 2 Differential expression of NK/T-cell effector and immune checkpoint markers between NTMT1-positive and NTMT1-negative epithelial–NK/T regions.

Supplementary Table 3 High-risk HPV genotyping results of the HSIL and cervical cancer specimens included in the present study.

Supplementary Figure 1 NTMT1 expression in cervical cancer and normal cervical tissues based on the TCGA-CESC and GTEx datasets.
