# Supplementary material for: NTMT1 (METTL11A) promotes MYC-dependent proliferation and downstream HLA-A suppression in cervical cancer
Source: Front Immunol. 2026 Jul 24;17:1853434. doi: 10.3389/fimmu.2026.1853434 (PMC13447250; doi:10.3389/fimmu.2026.1853434)
Supplement: Supplementary file 1 [file Table1.docx]

**Title: NTMT1 (METTL11A) couples MYC-driven proliferation with HLA-A–mediated immune evasion in cervical Cancer**

## Supplementary Table S1. Robustness of SCC findings across NTMT1-positivity thresholds.

| **Threshold** | **n_pos** | **% pos** | **Macrophage** | **NK/T** | **MYC** | **HLA-DRA** |
| --- | --- | --- | --- | --- | --- | --- |
| raw>0 (paper) | 579 | 33 | ↓ 6.2e-18 | ↑ 0.00073 | ↑ 0.033 | ↓ 0.0027 |
| raw>=2 | 180 | 10.2 | ↓ 6.9e-11 | ↓ 0.71 | ↑ 0.34 | ↓ 0.0053 |
| raw>=3 | 53 | 3 | ↓ 2.6e-05 | ↓ 0.89 | ↓ 0.71 | ↓ 0.00044 |
| top25% all spots | 440 | 25 | ↓ 2.3e-10 | ↑ 2.8e-07 | ↑ 0.5 | ↓ 0.073 |
| top10% all spots | 176 | 10 | ↓ 0.011 | ↑ 0.32 | ↓ 0.78 | ↑ 0.7 |
| >median of expressing | 289 | 16.4 | ↓ 1.5e-05 | ↑ 0.0025 | ↓ 0.96 | ↓ 0.35 |
| SCT-corrected>0 | 351 | 20 | ↓ 2.3e-07 | ↑ 0.004 | ↑ 0.065 | ↓ 0.48 |

*Direction (↑/↓) in NTMT1-positive vs negative spots and Wilcoxon p across alternative definitions of NTMT1-positivity.*

## Supplementary Table S2. NK/T effector and checkpoint markers, NTMT1⁺ vs NTMT1⁻ (epithelial–NK/T region).

| **Gene** | **mean NTMT1⁺** | **mean NTMT1⁻** | **log2FC** | **Wilcoxon p** | **adj. p** |
| --- | --- | --- | --- | --- | --- |
| GZMA | 0.357 | 0.334 | 0.034 | 0.29 | 0.57 |
| GZMB | 0.395 | 0.331 | 0.093 | 0.097 | 0.26 |
| GZMK | 0.138 | 0.145 | -0.009 | 0.81 | 0.95 |
| PRF1 | 0.368 | 0.283 | 0.123 | 0.019 | 0.08 |
| NKG7 | 0.513 | 0.555 | -0.061 | 0.61 | 0.89 |
| GNLY | 0.276 | 0.280 | -0.007 | 0.95 | 0.95 |
| IFNG | 0.108 | 0.058 | 0.073 | 0.0088 | 0.07 |
| PDCD1 | 0.077 | 0.023 | 0.078 | 0.00039 | 0.0063 |
| LAG3 | 0.358 | 0.279 | 0.113 | 0.02 | 0.08 |
| HAVCR2 | 0.188 | 0.214 | -0.037 | 0.39 | 0.69 |
| TIGIT | 0.144 | 0.153 | -0.014 | 0.94 | 0.95 |
| CTLA4 | 0.086 | 0.080 | 0.008 | 0.45 | 0.72 |
| TOX | 0.024 | 0.030 | -0.008 | 0.75 | 0.95 |
| BTLA | 0.016 | 0.017 | -0.000 | 0.92 | 0.95 |
| CD8A | 0.481 | 0.398 | +0.120 | 0.043 | 0.14 |
| CD3D | 0.281 | 0.240 | +0.059 | 0.2 | 0.45 |

*Mean SCT expression in NTMT1-positive vs NTMT1-negative spots of the epithelial–NK/T co-localization region, log2 fold-change, Wilcoxon p and BH-adjusted p. NTMT1-positive spots co-upregulate effector (PRF1, IFNG, CD8A) and inhibitory-checkpoint (PDCD1, LAG3) programs — a recruited-but-checkpoint-restrained signature.*

**Supplementary Table S3. HPV genotyping results of the HSIL and cervical cancer specimens included in this study.**

| Patient Subgroups | Pathology Number | HPV Genotyping |
| --- | --- | --- |
| Cervical Cancer | 23-61928 | 52 positive |
|  | 23-66766 | 16 positive |
|  | 25-114594 | 16 positive,18 positive |
| HSIL | Q25-1453 | 52 positive |
|  | Q23-01451 | 16 positive,18 positive,52 positive,81 positive |
|  | Q23-01800 | 16 positive,42 positive |

**Supplementary Figure S1. NTMT1 expression in cervical tumour versus normal cervix (TCGA + GTEx).**

*Note: NTMT1 (METTL11A) expression in TCGA-CESC primary tumours (n = 304) versus normal cervix (n = 13). Expression is modestly but significantly higher in tumour (~1.45-fold; two-sided Mann–Whitney p = 4.2×10⁻⁴).*
